# The Effect of GH Administration on Oocyte and Zygote Quality in Young Women With Repeated Implantation Failure After IVF

**DOI:** 10.3389/fendo.2020.519572

**Published:** 2020-09-29

**Authors:** Jan Tesarik, Maribel Galán-Lázaro, Cristina Conde-López, Agnese M. Chiara-Rapisarda, Raquel Mendoza-Tesarik

**Affiliations:** ^1^MARGen Clinic, Granada, Spain; ^2^Department of Obstetrics and Gynecology, University of Catania, Catania, Italy

**Keywords:** oocyte quality, embryo quality, implantation rate, live birth rate, growth hormome

## Abstract

Growth hormone (GH) has been shown to improve implantation and live birth rates in women of >40 years of age treated by *in vitro* fertilization (IVF). This effect was initially attributed to a GH effect on oocyte quality, but later studies showed that GH can also improve uterine receptivity for embryo implantation. As to younger women with previous failures of embryo implantation after IVF, data reported in the literature are ambiguous. This retrospective study focused on this latter category of women, comparing the numbers and morphological appearance of oocytes recovered from women with two previous IVF failures, aged between 30 and 39 years and treated with GH, with a comparable group of women without GH treatment. These results were complemented with the analysis of morphological markers of zygote and embryo quality and IVF clinical outcomes in both groups. The oocytes, zygotes and embryos from women treated with GH showed better morphological scores, and their uterine transfer resulted in more implantations, pregnancies and live births, as compared with the untreated group. It is concluded that the improvement of IVF outcomes in women with previous repeated IVF failures by exogenous GH administration is, at least partly, related to an increase in oocyte developmental potential. The statistically evident improvement of oocyte and embryo quality is the main finding of this study. Its weakness is its retrospective nature.

## Introduction

Exogenous growth hormone (GH) administration has been introduced to protocols of ovarian stimulation for *in vitro* fertilization (IVF) since the late 1980s and shown to improve IVF clinical outcomes (1–7), in agreement with observations on a positive relationship between GH concentration in follicular fluid aspirated from ovaries of patients treated by IVF and the treatment outcomes (8, 9). However, in spite of these encouraging initial data, some subsequent studies failed to find an improvement of IVF clinical outcomes after the inclusion of GH in the ovarian stimulation protocol (10, 11). These data suggest that GH treatment cannot improve IVF outcome in all patients with poor response to ovarian stimulation and open the question of how to identify patients who can benefit from this treatment.

There is solid evidence indicating that GH co-treatment during ovarian stimulation can enhance IVF outcomes in women aged >40 years (12, 13), but also in some younger women with previous repeated IVF failures (14), low response to high-dose stimulation (15, 16) and poor oocyte and embryo quality (14). Some studies have suggested an effect of GH on oocyte quality rather than quantity, through an improvement of cytoplasmic maturation with consequent reduction of aneuploidy caused by errors in the first and the second meiotic divisions (12, 14), while others also showed an effect on the number of retrievable oocytes, mediated by an increase in FSH- LH- and bone morphogenetic protein (BMP)- receptor density, as well as the density of its own receptors in granulosa cells, by GH treatment (13). Interestingly, a recent study reported an increase in the number of total retrieved, mature and fertilized oocytes, available embryos and high-quality embryos in all women with poor ovarian response treated with GH, independently of their age, but a significant increase in the implantation and pregnancy rate was found only in the older patients (17).

Moreover, the beneficial effects of GH administration on IVF outcomes, demonstrated in some patients, may not be caused solely by the hormone effect on the ovarian function. In fact, recent data have shown that the treatment with GH can also promote embryo implantation by improving uterine receptivity. This was demonstrated by two studies in which an effect of GH on oocyte quality could be excluded. Both studies used GH during the preparation of women for the transfer of embryos resulting from oocytes obtained in ovarian stimulation cycles not including GH administration. One dealt with transfers of the patients' own cryopreserved embryos resulting from a previous ovarian stimulation (18), and the other with transfers of fresh embryos from donated oocytes in patients with previous unexplained oocyte donation failure (19). Another study suggested that GH can both improve embryo quality and increase endometrial thickness in patients undergoing IVF, the former effect being more pronounced in women of <35 years of age and the latter in the older ones (17, 20). Altogether, the published data suggest that GH administration during ovarian stimulation can improve IVF outcomes in some, but not all, cases. It is not clear whether this effect is mainly due to the action of the hormone on oocyte quality or uterine receptivity, and how it is related to the patient's age. While the main cause of IVF failure in older women is supposed to be related with oocyte aneuploidy, mainly due to premature loss of centromeric cohesion between sister chromatids (21), and GH appears to alleviate this condition (12, 14), this may not be the case in younger women with poor response to ovarian stimulation treatments (22) in whom the mechanism of GH effect of IVF outcomes is even less clear.

This study was undertaken to evaluate the effects of GH administration during ovarian stimulation on the number of oocytes retrieved as well as on different morphological markers of oocyte and embryo quality and on IVF outcomes in young women with previous IVF failures.

## Results

In spite of the retrospective character of this study (see Materials and Methods), the patients treated and those not treated with GH were similar as to their age, duration of infertility, and basic parameters of ovarian function (Table 1).

**Table 1:** Baseline characteristics of women (*n* = 98) treated (*n* = 52) and untreated (*n* = 46) with GH.

**Treatment**	**Age (year)**	**BMI (kg/m^**2**^)**	**Infertility duration (year)**	**AFC (*n*)**	**Serum AMH (ng/ml)**
Without GH	34.5 ± 4.9	21.9 ± 4.2	3.9 ± 1.8	6.8 ± 4.1	2.3 ± 1.4
With GH	34.8 ± 4.1	22.2 ± 4.3	4.1 ± 2.1	6.4 ± 4.0	2.2 ± 1.5
*P*-value	>0.05	>0.05	>0.05	>0.05	>0.05

*Values are mean ± SD (%)*.

With the same protocol of ovarian stimulation, taking into account the individual condition of each patient and adjusted during the stimulation according the patient's response as described previously (23, 24), there was no difference in the total number of oocytes (Table 1) and of mature (metaphase II) oocytes (Table 2) retrieved from women co-stimulated with GH as compared with those in whom GH was not used. By contrast the number and percentage of oocytes with the best cumulative morphological quality score (Type A, Figure 1.1) was significantly higher, and those of oocytes with the worst score (Type D, Figure 1.4) was significantly lower in patients treated with GH as compared with the untreated patients (Table 2). As to the number and percentage of oocytes with the intermediate cumulative quality scores (Types B, Figure 1.2 and C, Figure 1.3), there was no difference between the two groups of patients (Table 2). No significant difference in endometrial thickness (*P* > 0.05) was detected between the protocols that included GH (9.0 ± 1.3 mm) and those that did not (8.8 ± 1.2 mm).

**Table 2:** Effect of GH treatment during ovarian stimulation on oocyte quality.

**Treatment**	**Metaphase II (MII) oocytes retrieved per patient**^****a****^				
	**Total MII oocytes**	**Type A**	**Type B**	**Type C**	**Type D**
Without GH	6.5 ± 3.2 (100)	2.9 ± 2.7 (45)	1.6 ±1.4 (25)	1.0 ± 1.3 (15)	1.0 ± 1.3 (15)
With GH	6.9 ± 2.9 (100)	4.1 ± 2.9 (59)	1.7 ± 1.7 (25)	0.8 ± 0.9 (12)	0.3 ± 0.6 (4)
*P* value	>0.05	<0.05	>0.05	>0.05	<0.01

a *Values are mean ± SD (%)*.

**Figure 1:**
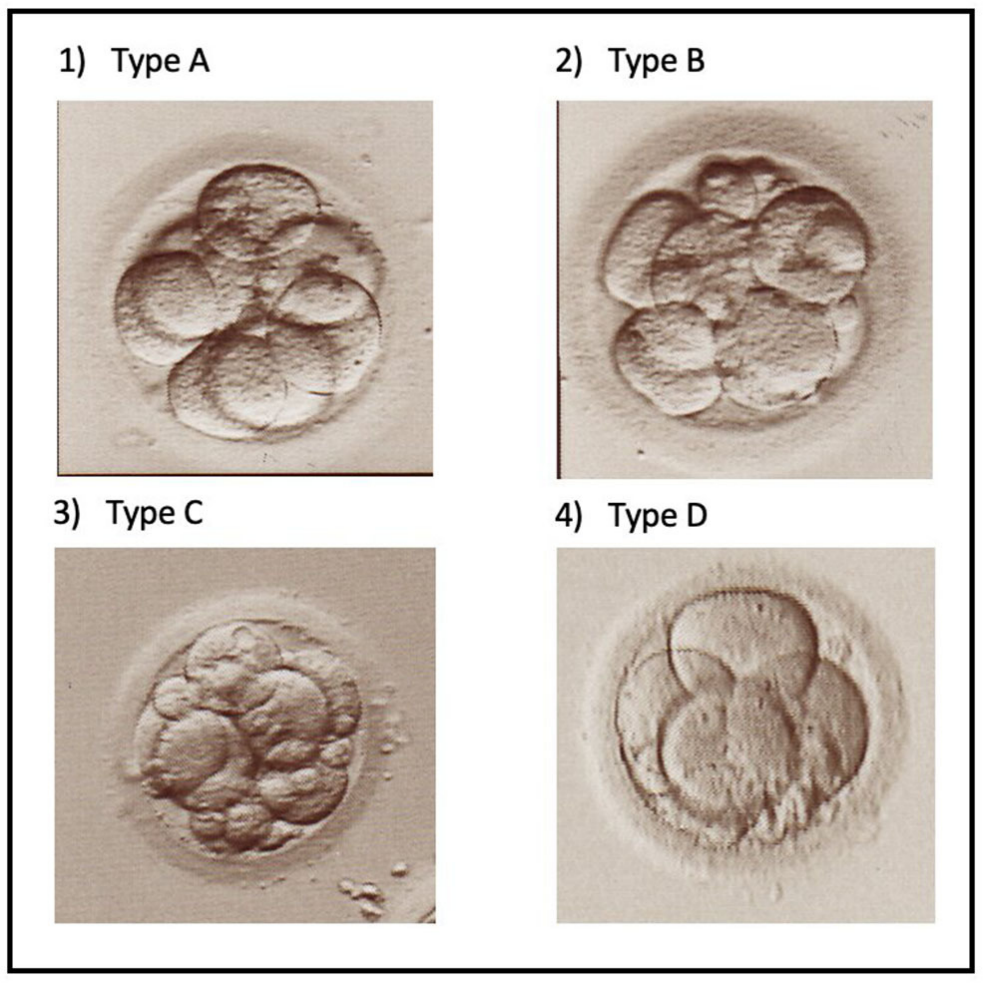
Micrographs showing different types of cleaving embryos on day 3 after ICSI. (1) Type A embryo with adequate number of cells of equal size (8) and only a few cell fragments. (2) Type B embryo with adequate number of cells (8) but some of unequal size, and a larger volume occupied by cell fragments. (3) Type C embryo with a lower number of cells (6) and numerous cell fragments. (4) Type D embryo apparently blocked at the 4-cell stage and some cell fragments.

Patients treated with GH had significantly more total zygotes and good-quality zygotes, according to the evaluation of pronuclear morphology (Table 3), more total cleaving embryos and those with the highest cumulative morphological quality score (Type A) and less embryos with the lowest scores (Types C and D) as compared with the untreated patients (Table 4). Like the oocyte quality scores (Table 2), there was no significant difference in the number and percentage of embryos with intermediate quality score (Type B) between both groups (Table 4).

**Table 3:** Effect of GH treatment during ovarian stimulation on zygote quality.

**Treatment**	**Zygotes with normal and abnormal pronuclear pattern achieved per patient**^****a****^		
	**Total**	**Normal pattern**	**Abnormal pattern**
Without GH	4.6 ± 2.5 (100)	0.6 ± 0.8 (13)	4.0 ± 2.0 (87)
With GH	5.8 ± 2.5 (100)	1.4 ± 1.2 (24)	4.4 ± 2.4 (76)
*P*-value	<0.05	<0.01	>0.05

a *Values are mean ± SD (%)*.

**Table 4:** Effect of GH treatment during ovarian stimulation on the quality of embryos achieved.

**Treatment**	**Embryos achieved per patient**^****a****^				
	**Total**	**Type A**	**Type B**	**Type C**	**Type D**
Without GH	4.7 ± 2.5 (100)	1.4 ± 1.2 (30)	1.7 ± 1.6 (36)	1.0 ± 0.8 (21)	0.6 ± 0.8 (13)
With GH	5.9 ± 2.4 (100)	3.2 ± 2.1 (54)	1.9 ± 1.4 (32)	0.6 ± 0.8 (10)	0.0
*P*-value	<0.05	<0.01	>0.05	<0.05	<0.05

a *Values are mean ± SD (%)*.

Similar numbers of embryos were transferred in patients treated and in those untreated with GH, but the patients of the former group received more high-quality embryos as compared with those of the latter (Table 5). More patients treated with GH became pregnant after embryo transfer, and developed more gestational sacs, as compared with the untreated patient group (Table 6). Consequently, both pregnancy rate and implantation rate were significantly improved by GH administration (Table 6).

**Table 5:** Effect of GH treatment during ovarian stimulation on the quality of embryos transferred.

**Treatment**	**Embryos transferred per patient**^****a****^			
	**Total**	**Type A**	**Type B**	**Type C**
Without GH	2.4 ± 0.6 (100)	1.0 ± 1.1 (42)	0.9 ± 0.7 (37)	0.5 ± 0.7 (21)
With GH	2.2 ± 0.6 (100)	1.8 ± 0.8 (82)	0.3 ± 0.6 (14)	0.1 ± 0.1 (4)
*P*-value	>0.05	<0.05	<0.01	<0.01

a *Values are mean ± SD (%)*.

**Table 6:** Effect of GH treatment on clinical pregnancy and delivery rate.

**Treatment**	**Embryo transfers**	**Clinical pregnancies**	**Deliveries**	**Pregnancy rate**	**Delivery rate**
Without GH	46	5	3	10.9%	6.5%
With GH	52	22	18	42.3%	34.6%
*P*-value				<0.01	<0.01

Eighteen healthy babies were born in patients treated with GH, as opposed to only 3 in the untreated patient group, marking a significant difference in both the delivery rate (Table 6) and birth rate (Table 7) in favor of the GH-treated patient group.

**Table 7:** Effect of GH treatment on clinical implantation and birth rate.

**Treatment**	**Embryo transferred**	**Gestational sacs with heartbeat**	**Babies born**	**Clinical implantation rate**	**Birth rate**
Without GH	110	5	3	4.5%	2.7%
With GH	104	22	18	21.2%	17.3%
*P*-value				<0.01	<0.01

No complications were observed in either group of patients during and after ovarian stimulation.

## Discussion

The present data show that, independently of eventual effect on uterine receptivity, GH has a clear beneficial influence on the quantity and morphological quality of oocytes zygotes and cleaving embryos when administered to young women with previous IVF failures. These improvements are accompanied by a significant increase in the clinical pregnancy, delivery, implantation and birth rates in this group of patients. No multiple pregnancy was established in either of the two groups, which is somewhat surprising especially in the GH group. Though this might be a matter of chance, it is also possible that some of the patients had other predisposing factors for implantation failure, not resolvable by GH treatment.

Unlike the study by Jin et al. (17), the number of mature (metaphase II) oocytes retrieved in patients treated and those untreated with GH was similar. However, in agreement with those previous observations (17), there were more good-quality oocytes, fertilized oocytes and good-quality zygotes and embryos in the GH group. GH administration was shown to enhance FSH- LH- and bone morphogenetic protein (BMP)- receptor density in ovarian follicular granulosa cells (13). This effect may lead to an increase in the number of retrievable oocytes in women with poor ovarian response (13, 17), whereas it may improve the quality rather than the quantity of oocytes recovered from women with basically normal ovarian response. This kind of patients was prevalent in the present study. This hypothesis is further substantiated by the present observation that GH administration in this group of patients did not only improve the morphological quality of the oocytes obtained, but it also increased the total number of fertilized oocytes (zygotes), that of normal zygotes and that of high-quality embryos, as judged by their morphological appearance. Among these characteristics, that of the zygote quality seems to be of particular importance, since it was previously shown that zygote pronuclear morphology is related not only with IVF clinical outcomes (25–28) and the rate of embryo development to the blastocyst stage (27), but also with the normal ploidy of the resulting blastocysts (29–31). When used in combination with further evaluation of embryo morphology during subsequent stages of preimplantation development, the prognostic value of zygote morphology, as to the probability of establishing a normal pregnancy, was further enhanced (32, 33).

This study showed a significant improvement of both zygote and cleaving embryo morphology by the administration of GH during ovarian stimulation. This suggests that the improvement of oocyte quality is an important mechanism of action of GH responsible for the improvement of pregnancy, implantation, delivery and live birth rates in young women with previous IVF failures. If confirmed, the up-regulation of granulosa cell receptors by GH may be involved in oocyte cytoplasmic maturation which, in its turn, may stabilize the function of cohesin and other key proteins involved in the correct function of the meiotic spindle during the final phases of oocyte nuclear maturation. An effect of GH on embryo implantation may have also acted as an independent factor in some of these cases, but it appears to be marginal as compared with the effect on oocyte quality, in agreement with the previous observation of a relatively low prevalence of cases with repeated implantation failures after oocyte donation resolved by GH administration to oocyte recipients (19).

The mechanism through which exogenous GH can improve oocyte quality in young women remains to be elucidated. It may be related to the previously reported increase in the density of receptors for FSH, LH, BMP receptor 1B, as well as its own receptor (13). However, the above observations were obtained with an older patient population as compared with that involved in our study. It remains to be determined whether GH produces similar effects in younger women with previous IVF failures, supposedly related to poor oocyte quality. It remains to be determined whether the beneficial effects of GH on oocyte quality are mainly mediated by a direct action through its own receptors or by an increase in the secretion of IGF-1. Studies are in progress to address these questions in order to characterize better those women who are likely to benefit from GH co-stimulation to improve IVF outcomes. In addition to the effect on oocyte quality, improvement of uterine receptivity (19) may also have contributed to the positive effects of GH on embryo implantation in some patients, although no difference in endometrial thickness was found between patients who were treated with GH and those who were not. However, endometrial receptivity is not necessarily reflected by endometrial thickness, and the design of this study does not allow to discriminate between these two mechanisms. This would only be possible with an oocyte donation model.

It also remains to be determined why GH administration has more effect in some young women than in others. While this paper was under review, we have addressed specifically this question, with another group of patients. We found that some young women have their “GH-age,” determined indirectly by measuring their serum IGF-1 concentrations (GH is too fluctuating to give a reliable result) up to 20 years above their chronological age (34). This was not done in the women included in the present study. It seems that externally administered GH has less effect in young women with normal intrinsic GH production (34).

## Materials and Methods

### Study Design, Participants, and Their Allocation to Groups

This retrospective study was approved by the ethical committee of our clinic. All procedures performed in this study were in accordance with the ethical standards of the institutional and national research committee and with the 1964 Helsinki declaration and its later amendments. All participants signed an informed consent. The study involved 98 women, aged between 30 and 39 years, and having undergone at least 2 previous unsuccessful IVF attempts in spite of generating acceptable numbers of oocytes and embryos. They were considered for a new treatment attempt at the MARGen Clinic in the period between January 2014 and December 2017. Fifty-two of these women were treated with GH during ovarian stimulation, whereas the other 46 were not. The patient allocation to each of the two groups was based on the couples' own decision after having received exhaustive information concerning the current knowledge about the use of GH in their situation.

In fact, our ethical committee discouraged a randomized controlled trial because, in view of our previous results, the deliberate allocation of patients to the control group may cause harm. Thus, all the pros and cons, as well as the lack of solid evidence in favor of GH were thoroughly discussed with each couple. The decision was then taken by the couples, not by the medical staff.

It was explained that GH has been shown clearly to improve IVF outcomes in older women, but its benefits for younger women undergoing IVF treatment is controversial. Since the short treatment with GH has no side-effects on the patient's health, the patients' decision as to the use of this treatment was sometimes motivated by its cost. Some patients also preferred not to be included in the GH group because of concern about potentially useless “overmedication,” adding more daily injections to the already quite complex ovarian stimulation protocol. In spite of the absence of any artificial “matching,” patients who decided to be included in the GH group had similar baseline characteristics as compared to those who preferred the standard ovarian stimulation protocol (Table 1). But for the exclusion of GH administration from the ovarian stimulation protocol, these patients were treated exactly as those of the GH group. Only couples with normal basic sperm parameters and normal percentage of spermatozoa with DNA fragmentation (35) were included.

### Assisted Reproductive Technologies (ART)

IVF was performed by intracytoplasmic sperm injection (ICSI) in all patients involved in this study, after ovarian stimulation using a GnRH antagonist protocol. Details of both the clinical and the laboratory protocols used were published in detail previously (19). Briefly, patients were treated by daily injections of recombinant follicle-stimulating hormone (FSH) (Puregon or Gonal F) and human menopausal gonadotropin (HMG) (Menopur), started between the second and the fourth day following the beginning of menstrual bleeding. The initial doses of FSH and HMG were determined according to the markers of the patients' ovarian reserve, antral follicle count and serum concentration of LH on the day preceding the beginning of stimulation. If serum LH level before the beginning of ovarian stimulation was below 2 IU/L, HMG was added to FSH. If the small antral follicle count in both ovaries was equal to or higher than 10 and the serum LH concentration was between 1 and 2 IU/l, the usual daily dose of HMG during the first 4 days of stimulation was 75 IU. When there were <10 small antral follicles in both ovaries, the usual dose of HMG was 150 IU. The HMG treatment was always accompanied by FSH whose dose was adapted according to the patient's basal serum anti-Mullerian hormone concentration. The following ultrasound examinations, as well as the determinations of serum estradiol and LH concentrations were done on the 5th day of stimulation and then every other day until the administration of ovulation trigger. The respective doses of FSH and HMG administered were adapted, in a flexible manner, according to the results of each of these examinations, in the same way as described for the long GnRH agonist-controlled ovarian stimulation protocol (19). Briefly, FSH dose was basically determined according to serum estradiol concentration and the number and size of antral follicles. If serum LH concentration tended to decrease, especially after the onset of GnRH antagonist treatment, the growth of all follicles was slow, and no tendency for dominance was observed, higher doses of HMG (75–150 IU) were maintained. If, on the other hand, serum LH concentrations increased, follicular growth was rapid and some follicles grew more rapidly than others, HMG was maintained at minimal doses or even withdrawn, finishing the whole ovarian stimulation procedure with FSH alone. If different, and sometimes opposite, tendencies in all these parameters were observed, the clinician took the decision after pondering the advantages and disadvantages of different FSH-to-HMG dose ratios, taking into consideration the history and the complete clinical picture of each case. Ovulation was triggered by subcutaneous injection of 250 μg recombinant human chorionic gonadotropin (HCG; Ovitrelle) when at least two follicles reached the size of 17–18 mm. Ovarian puncture for oocyte recovery was performed 36.5 h after the HCG injection.

In spite of the fact that all male partners had normal sperm parameters, the high-magnification ICSI, also called intracytoplasmic morphologically selected sperm injection (IMSI) was used in all cases by precaution, taking into account the history of the patients' previous IVF failures, as described previously (36). All embryos were transferred on Day 3 after ICSI. At least one embryo of acceptable quality (cumulative scores A, B or C, excluding score D) was available for transfer in all cases. Luteal phase was supported with intravaginal micronized progesterone, beginning on the day of oocyte recovery, at daily doses ranging between 200 and 600 mg, according to serum progesterone concentration, determined on the day of embryo transfer and then every 7 days during the first month after the transfer.

### Protocol of GH Administration

A total dose of 10 mg GH (Nutropin) was administered in 10 daily doses of 1 mg, starting on the first day of ovarian stimulation. When the ovarian stimulation was shorter than 10 days, the rest of the total 10-mg dose was administered on the day following the application of ovulation trigger. This short GH administration protocol was based on our previous work (12) aimed at improving oocyte quality rather than quantity. In fact, the women included in this study were relatively young and yielded sufficient numbers of oocytes in their previous unsuccessful treatment attempts.

### Evaluation of Oocyte, Zygote, and Embryo Quality

Oocyte, zygote and embryo quality were evaluated by microscopical examination using an inverted microscope (Olympus IX71) equipped with Hofman modulation contrast optics. Oocytes were attributed one of the four cumulative scores, from A (the best one) to D (the worst one), taking into account the oocyte shape, cytoplasmic granularity, the presence of intracytoplasmic vacuoles, the form of the zona pellucida and the perivitelline space, and the size and morphology of the first polar body (37). Zygotes and cleaving embryos (Figure 1) were scored as described previously (25–28, 32). Briefly, cleaving embryos were scored according to the number of cells, the shape and regularity of the cells, and the volume occupied by anucleate fragments (Figure 1).

### Statistical Analysis

Analysis was performed using SPSS version 16.0 for Microsoft Office (SPSS Inc., Chicago, IL, USA). The data were expressed as mean ± SD for quantitative variables or percentage (%) of or qualitative ones. Data between the two groups were analyzed by Student's *t*-test for quantitative variables and X2 tests for qualitative ones. All tests were two-tailed and a *P* < 0.05 was considered statistically significant.

## Conclusion

Independently of eventual contribution to an improvement of uterine receptivity, GH administration during ovarian stimulation of young women with previous IVF failures was clearly shown to improve IVF outcomes by increasing oocyte, zygote and embryo quality.

## Data Availability Statement

The datasets generated for this study are available on request to the corresponding author.

## Ethics Statement

The studies involving human participants were reviewed and approved by Foundation MARGen Mendoza Tesarik. Granada, Spain. The patients/participants provided their written informed consent to participate in this study.

## Author Contributions

RM-T and JT: conceptualization, validation, formal analysis, and writing-review and editing and writing original draft preparation. RM-T, MG-L, and CC-L: laboratory work and validation JT: clinical evaluation. RM-T: software. All authors contributed to the article and approved the submitted version.

## Conflict of Interest

The authors declare that the research was conducted in the absence of any commercial or financial relationships that could be construed as a potential conflict of interest.

## Abbreviations

GH: Growth hormone
IVF: *In vitro* fertilization
FSH: Follicle-stimulating hormone
LH: Luteinizing hormone
BMP: Bone morphogenetic protein
ICSI: Intracytoplasmic sperm injection
IMSI: Intracytoplasmic morphologically-selected sperm injection
GnRH: Gonadotropin releasing hormone
HMG: Human menopausal gonadotropin
HCG: Human chorionic gonadotropin
SD: Standard deviation.
